# Phylogenetic Position of *Haemaphysalis kashmirensis* and *Haemaphysalis cornupunctata*, with Notes on *Rickettsia* spp.

**DOI:** 10.3390/genes14020360

**Published:** 2023-01-30

**Authors:** Shah Masood Khan, Mehran Khan, Abdulaziz Alouffi, Mashal M. Almutairi, Muhmmad Numan, Shafi Ullah, Muhammad Kashif Obaid, Zia Ul Islam, Haroon Ahmed, Tetsuya Tanaka, Abid Ali

**Affiliations:** 1 Department of Zoology, Abdul Wali Khan University Mardan, Mardan 23200, Pakistan; 2 King Abdulaziz City for Science and Technology, Riyadh 12354, Saudi Arabia; 3 Department of Pharmacology and Toxicology, College of Pharmacy, King Saud University, Riyadh 11451, Saudi Arabia; 4 Department of Biotechnology, Abdul Wali Khan University Mardan, Mardan 23200, Pakistan; 5 Department of Biosciences, COMSATS University Islamabad (CUI), Park Road, Chak Shahzad, Islamabad 45550, Pakistan; 6 Laboratory of Infectious Diseases, Joint Faculty of Veterinary Medicine, Kagoshima University, Kagoshima 890-0065, Japan

**Keywords:** *Haemaphysalis kashmirensis*, *Haemaphysalis cornupunctata*, *Haemaphysalis montgomeryi*, *Rickettsia hoogstraalii*

## Abstract

Despite high diversity in the Oriental region, ticks of the genus *Haemaphysalis* have been neglected regarding their genetic data and vector potential. This study aimed to genetically characterize three species of the genus *Haemaphysalis*: *Haemaphysalis cornupunctata*, *Haemaphysalis kashmirensis,* and *Haemaphysalis montgomeryi* infesting goats and sheep, and *Rickettsia* spp. associated with these tick species in the Hindu Kush Himalayan range of Pakistan. Altogether, 834 ticks were collected by examining 120 hosts including goats (64/120, 53.3%) and sheep (56/120, 46.6%), in which 86 (71.6%) hosts were found to be tick-infested. The morphologically identified ticks were subjected to DNA extraction and PCR for the amplification of partial 16S rDNA and *cox* fragments. *Rickettsia* spp. associated with the collected ticks were detected through the amplification of *gltA*, *ompA* and *ompB* partial fragments. The 16S rDNA of *H. cornupunctata* and *H. montgomeryi* showed a maximum identity of 100% with the sequences of the same species, whereas the 16S rDNA of *H. kashmirensis* showed the highest identity of 93–95% with *Haemaphysalis sulcata*. The *cox* sequence of *H. montgomeryi* displayed 100% identity with the same species. In comparison, the *cox* sequences of *H. cornupunctata* and *H. kashmirensis* showed maximum identities of 87.65–89.22% with *Haemaphysalis punctata* and 89.34% with *H. sulcata*, respectively. The *gltA* sequence of *Rickettsia* sp. from *H. kashmirensis* showed the highest identity of 97.89% with *Rickettsia conorii* subsp. *raoultii*, while the *ompA* and *ompB* fragments from the same DNA samples revealed 100% and 98.16% identity with *Rickettsia* sp. and “*Candidatus* Rickettsia longicornii”, respectively. Another *gltA* sequence amplified from *H. montgomeryi* ticks showed 100% identity with *Rickettsia hoogstraalii*, while the attempts to amplify *ompA* and *ompB* for *R. hoogstraalii* were unsuccessful. In the phylogenetic tree, the 16S rDNA of *H. cornupunctata* clustered with the corresponding species while its *cox* clustered with *H. punctata*. Both 16S rDNA and *cox* sequences of *H. kashmirensis* clustered with *H. sulcata*. The *gltA* sequence of *Rickettsia* sp. was clustered individually in the spotted fever (SF) group of *Rickettsia*, while the *gltA* sequence of *R. hoogstraalii* was clustered with the same species in the transition group of *Rickettsia*. In the SF group, the rickettsial *ompA* and *ompB* sequence clustered with undetermined *Rickettsia* sp. and “*Candidatus* Rickettsia longicornii”, respectively. This is the earliest study regarding the genetic characterization of *H. kashmirensis*. This study indicated that ticks belong to the genus *Haemaphysalis* have the potential of harboring and/or transmitting *Rickettsia* spp. in the region.

## 1. Introduction

Ticks are notorious ectoparasites infesting the majority of terrestrial and semiterrestrial vertebrates [1]. Ticks are cosmopolitan in distribution, particularly prevalent in tropical and subtropical regions [1]. In addition to anemia, a reduction in dairy products and meat, ticks can also transmit numerous infectious agents including viruses, bacteria, protozoa, and helminths to vertebrate hosts [2]. 

*Haemaphysalis* (Acari: Ixodidae) is the second largest genus of ixodid ticks comprising approximately 176 species [1]. *Haemaphysalis* ticks inhabit various landscapes mainly in the Oriental, Afrotropical, and Palearctic regions, while some of its members have been recorded in Australasia (Australia, New Zealand, and New Guinea), infesting a variety of free-roaming and domestic animals [1,3]. Some species of *Haemaphysalis* have been identified as potential vectors for various protozoan, viral, and bacterial pathogens to humans, domestic, and wild animals [4,5]. *Haemaphysalis* ticks such as *Haemaphysalis longicornis, Haemaphysalis concinna, Haemaphysalis qinghaiensis,* and *Haemaphysalis flava* have been associated with various *Rickettsia* spp. including *Rickettsia japonica, R. conorii, Rickettsia monacensis, Rickettsia heilongjiangensis,* and several undetermined *Rickettsia* spp. [6,7,8,9]. Moreover, the number of *Rickettsia* spp. associated with *Haemaphysalis* ticks are continuously increasing because of the advancement in molecular approaches [10].

Important *Haemaphysalis* ticks such as *H. kashmirensis*, *H. cornupunctata,* and *H. montgomeryi* belong to the subgenus *Herpetobia*, *Aboimisalis*, and *Segalia*, respectively [11]. These tick species have been described by Hoogstraal and Varma (1962). Additionally, the *H. kashmirensis* and *H. cornupunctata* ticks were collected from Kashmir, while *H. montgomeryi* from India [12]. Later on, life stages such as larva and nymph of these ticks were also described [12,13]. *Haemaphysalis kashmirensis*, *H. cornupunctata,* and *H. montgomeryi* inhabit in the Oriental and Palearctic regions [1]. In the Indian subcontinent, the *H. cornupunctata*, *H. kashmirensis,* and *H. montgomeryi* have been reported from different regions of India, Kashmir, Nepal, and Pakistan, particularly located in the Hindu Kush Himalayan (HKH) range [14,15,16,17,18].

The livestock hosts in Pakistan are known to be infested by a variety of *Haemaphysalis* ticks [16,19,20]. Genetic characterization of closely related *Haemaphysalis* ticks such as *H*. *kashmirensis* and *H. cornupunctata* is important for the surveillance and control of these ticks in the Oriental region including Pakistan, which is an epidemic hotspot for these parasites [14,16,18]. The capabilities of *Haemaphysalis* ticks as a vector for *Rickettsia* spp. have been neglected. These ticks need to be molecularly screened for associated *Rickettsia* spp. This study was aimed to the molecular characterization of *H*. *kashmirensis* and *H. cornupunctata* ticks infesting goats and sheep, and associated *Rickettsia* spp. in the HKH range of Pakistan.

## 2. Materials and Methods

### 2.1. Study Sites

**Figure 1:**
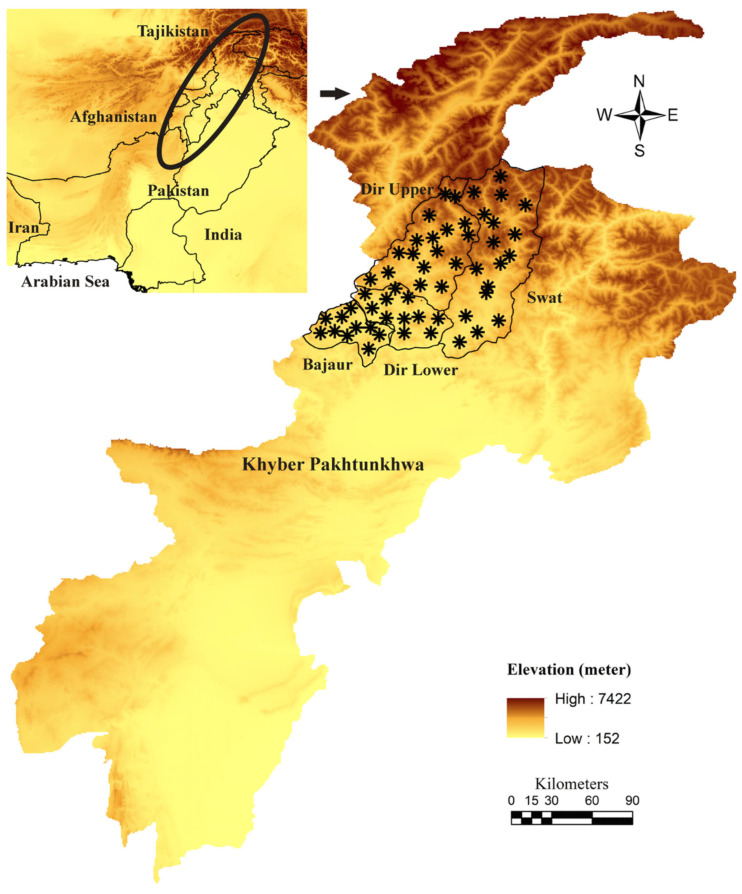
Elevation-based map showing tick collection sites (black asterisk).

The present study was conducted in four districts, namely: Dir Lower (34°52′12.1” N, 71°49′00.8” E), Dir Upper (35°12′30.06” N, 71°52′31.22” E), Bajaur (34°44′4.95” N, 71°30′47.80” E), and Swat (34°45′0.8634” N, 72°21′26.42” E) of the HKH ranges of Khyber Pakhtunkhwa (KP), Pakistan. The geographical coordinates of the tick collection sites were noted by Global Positioning System (GPS) and used for the designing of a map through ArcGIS 10.3.1 (ESRI, Redlands, CA, USA) (Figure 1).

### 2.2. Ethical Approval and Consent 

The design of the present study was approved by the Advanced Study and Research Board (Dir/A&R/AWKUM/2022/9396) and the Ethical Committee of the Faculty of Chemical and Life Sciences, Abdul Wali Khan University Mardan, Pakistan. All animal owners were informed verbally, and approval was taken before observing their hosts. 

### 2.3. Tick Collection and Morphological Identification

Villages of the four selected districts were randomly visited between March 2021 to February 2022 for tick collection. Small ruminants including goats and sheep were examined for ticks. When found, ticks from each host were separately collected in labeled tubes using a fine tweezer. The necessary information regarding collection sites, host type and gender, collection date, and environmental conditions (temperature and humidity) were noted. Collected ticks were rinsed with distilled water followed by 70% ethanol to remove contaminants. Subsequently, standard identification keys [12,13,14,21] were used to identify tick species based on observation of morphological features such as shape of capitulum, cornua and palp articles, cervical and lateral grooves, size and shape of coxa spurs, number of festoons, and shape of genital aperture. These morphological observations were performed under a stereomicroscope (SZ61, Olympus Corporation, Tokyo, Japan).

### 2.4. Statistical Analyses

Necessary tick data recorded from the four districts were compiled and arranged in spreadsheets using Microsoft Excel V. 2016 (Microsoft Office 365^®^). Prevalence (no. of infested hosts×100/total no. of examined hosts), mean abundance (total no. of collected ticks/total no. of examined hosts), and mean intensity (total no. of collected ticks/no. of infested hosts) was determined. The necessary climate data for each month or seasonal were obtained via mean temperature (°C), relative humidity (%), and total rainfall (mm) (climate-data.org; accessed on 15 November 2022). 

### 2.5. DNA Extraction and PCR Amplification

Altogether, 108 tick specimens (three males, three females, and three nymphs per species per district) were randomly selected for molecular analysis. The morphologically identified ticks were individually dissected with a sterile blade and ground with a hygienic pestle in 1.5 mL Eppendorf tubes. The ground samples were individually subjected to the phenol–chloroform protocol [22] for DNA extraction. The extracted genomic DNA was quantified by using NanoDrop (Nano-Q, Optizen, South Korea).

The extracted DNA was subjected to conventional PCR to amplify 16S rDNA (460 bp) and *cox* (710 bp) fragments of tick species and citrate synthase (*gltA*), outer membrane protein subunit A (*ompA*), and outer membrane protein subunit B (*ompB*) fragment for any *Rickettsia* spp. Each PCR reaction comprised 25 µL volume: 8.5 µL of PCR water “nuclease free”, 1 µL of each primer at a concentration of 10 pmol/µL, 2 µL of extracted DNA (50–100 ng/µL), and 12.5 µL Dream*Taq* green MasterMix (2X) (Table 1). In each PCR reaction, PCR water was used as a negative control while *Rhipicephalus microplus* and *Rickettsia massiliae* DNA were taken as positive control for ticks and *Rickettsia* spp., respectively. PCR products were analyzed by horizontal electrophoresis in 2% agarose gel and examined under ultraviolet light of a Gel Documentation System (BioDoc-It™ Imaging Systems, Upland, CA, USA). 

### 2.6. Sequencing and Phylogenetic Analysis

Amplicons were purified with GeneClean II Kit (Qbiogene, Illkirch, France) following the manufacturer’s protocol and sequenced bi-directionally (Macrogen, Inc., Seoul, South Korea) via the Sanger-based sequencing method. The obtained sequences were trimmed by removing the poor-quality regions followed by obtaining a consensus sequence in SeqMan v 5.00 (DNASTAR, Inc., Madison, WI, USA). Maximum identity sequences were retrieved from GenBank using the Basic Local Alignment Search Tool (BLAST) [28] on the National Center for Biotechnology Information (NCBI) user interface. They were aligned with the obtained sequences in BioEdit Sequence Alignment Editor v. 7.0.5 [29] using CLUSTAL W multiple alignments [30]. The Neighbor-Joining method with the Kimura 2-parameter model was applied to construct phylogenetic trees with 1000 bootstrap replicates in Molecular Evolutionary Genetic Analysis (MEGA-X) software [31]. The coding (*cox*, *gltA*, *ompA,* and *ompB*) nucleotide sequences were aligned by MUSCLE [32]. The final positions in the dataset comprised the obtained sequence of each fragment. 

## 3. Results

### 3.1. Ticks, Hosts, and Seasonal Data

Altogether, 834 tick specimens belonging to three tick species including *H. cornupunctata* (258, 30.9%: 145 females, 86 males, 27 nymphs), *H. kashmirensis* (191, 22.8%: 102 females, 67 males, 22 nymphs), and *H. montgomeryi* (385, 46.2%: 202 females, 148 males, 35 nymphs) were morphologically identified. The details about the ticks’ prevalence and their life stages in each district are provided in Table 2.

**Figure 2:**
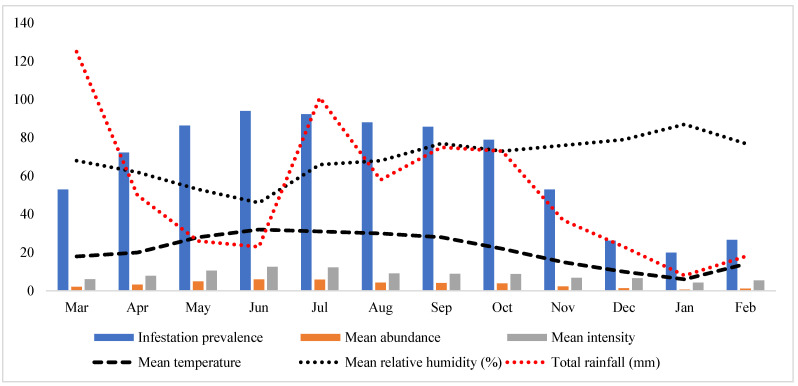
The graph shows the variation in prevalence, mean abundance, and mean intensity related to mean temperature, mean relative humidity, and the total rainfall during this study.

Ticks were collected from 86 out of 120 examined hosts (goats and sheep) with an overall 71.3% prevalence. Goats were observed with a high prevalence (49/64, 76.6%) compared to sheep (37/56, 66.1%). An overall mean abundance of 7.0 ticks/examined host was noted, while the mean intensity was 9.7 ticks/infested host. Goats were found with higher mean abundance and mean intensity (8.1 and 10.6, respectively) compared to sheep (5.6 and 8.6, respectively). Seasonal parameters, including temperature, humidity, and rainfall with tick parameters such as the prevalence of infestation, mean abundance, and mean intensity are shown in Figure 2.

### 3.2. Molecular Analysis

Altogether, 72 sequences were obtained (one 16S rDNA and one *cox* for one female, one male, and one nymph of each tick species) for ticks. Trimmed sequences for *H*. *cornupunctata*, *H*. *kashmirensis,* and *H*. *montgomeryi* were 16S rDNA (395 bp) and *cox* (654 bp). The 16S rDNA of *H*. *cornupunctata* showed 100% identity with the same species from Pakistan. In contrast, its *cox* showed 87.65–89.22% identity with the same subgenus species: *H.* (*Aboimisalis*) *punctata* from Portugal, Iran, France, China, Hungary, and Romania. The BLAST outcome for the 16S rDNA of *H*. *kashmirensis* revealed 93–95% identity with the same subgenus species: *H.* (*Herpetobia*) *sulcata* reported from France, Turkey, China, and Pakistan, compared to *cox* which showed 89.34% identity with *H. sulcata* reported from China and Iran. The sequences obtained for *H. montgomeryi* were 100% identical to the sequences of the same species reported from Pakistan [16]. Thus, such sequences were excluded from further analysis.

For *Rickettsia* spp., bidirectional sequences were obtained for each amplified fragment. The consensus sequences of *gltA* (380 bp), *ompA* (504 bp), and *ompB* (449 bp) were amplified from *H*. *kashmirensis,* while *gltA* (340 bp) was amplified from *H*. *montgomeryi*. Rickettsial *gltA* sequence (380 bp) amplified from *H*. *kashmirensis* showed 97.89% identity with *R. conorii* subsp. *raoultii* from Brazil, China, and France, while another rickettsial *gltA* consensus sequence from *H*. *montgomeryi* displayed 100% identity with *R. hoogstraalii* reported from Italy. The *ompA* amplified from the same samples of *H*. *kashmirensis* showed 100% identity with *Rickettsia* sp. from China and Algeria. The *ompB* sequence of the corresponding sample showed 98.16% identity with “*Candidatus* Rickettsia longicornii” reported from China. The rate of infection was 15.74% (17/108) recorded for *Rickettsia* sp. (based on *gltA, ompA,* and *ompB)* followed by 5.56% (6/108) for *R. hoogstraalii* (based only on *gltA*). The details regarding the infection rate and the number of sequences obtained for each *Rickettsia* spp. are shown in Table 2.

The obtained 16S rDNA sequences were submitted to GenBank under the accession numbers: OQ024373 (*H. cornupunctata*) and OQ024650 (*H. kashmirensis*); *cox* sequences under the accession numbers: OQ096502 (*H. cornupunctata*) and OQ096625 (*H. kashmirensis*); rickettsial *gltA* sequences under the accession numbers: OQ160793 (*Rickettsia* sp.) and OQ160792 (*R. hoogstraalii*); rickettsial *ompA* sequence under the accession number: OQ108505 (*Rickettsia* sp.), while rickettsial *ompB* sequence under the accession number: OQ055189 (*Rickettsia* sp.).

### 3.3. Phylogenetic Analysis 

**Figure 3:**
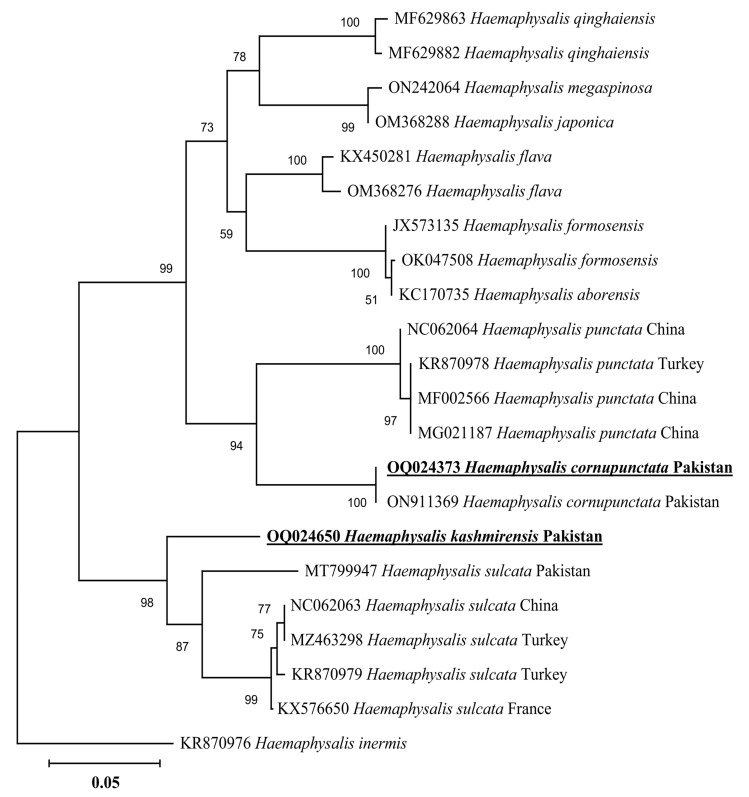
Neighbor-Joining phylogenetic tree based on 16S rDNA of *H. cornupunctata* and *H. kashmirensis*. The 16S rDNA sequence of *Haemaphysalis inermis* was employed as an outgroup. All sequences have been denoted by their GenBank accession numbers, followed by species name and country name. The bootstrap values (1000-replications) are shown at each node. The sequences (OQ024373 and OQ024650) of the present study have been marked with bold and underlined fonts.

**Figure 4:**
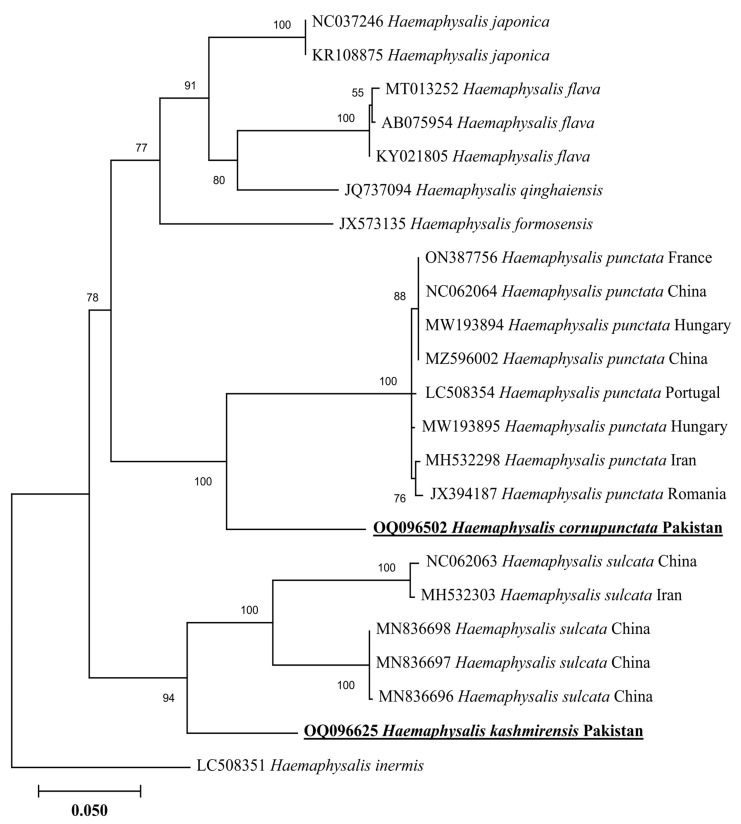
Neighbor-Joining phylogenetic tree based on *cox* of *H. cornupunctata* and *H. kashmirensis*. The *cox* sequence of *H. inermis* was employed as an outgroup. All sequences have been denoted by their GenBank accession numbers, followed by species name and country name. The bootstrap values (1000-replications) are shown at each node. The sequences (OQ096502 and OQ096625) of the present study have been marked with bold and underlined fonts.

In a phylogenetic tree based on 16S rDNA, the *H. cornupunctata* sequence clustered with the same species reported from Pakistan (ON911369), whereas this species grouped in a sister clade with the species of the same subgenus: *H*. *punctata* reported from China (NC062064, MF002566, and MG021187) and Turkey (KR870978) (Figure 3). The 16S rDNA sequence of *H. kashmirensis* clustered with the species of the same subgenus: *H.* (*Herpetobia*) *sulcata* reported from France (KX576650), Turkey (MZ463298 and KR870979), China (NC062063), and Pakistan (MT799947) (Figure 3). In a phylogenetic tree based on *cox*, the *H. cornupunctata* sequence clustered with the species of the same subgenus: *H*. *punctata* reported from Portugal (LC508354), Iran (MH532298), France (ON387756), China (NC062064 and MZ596002), Hungary (MW193894-MW193895), and Romania (JX394187) (Figure 4). Whereas the obtained *cox* sequence of *H. kashmirensis* sequence clustered with the species of the same subgenus: *H.* (*Herpetobia*) *sulcata* reported from China (MN836696-MN836698 and NC062063) and Iran (MH532303) (Figure 4). 

**Figure 5:**
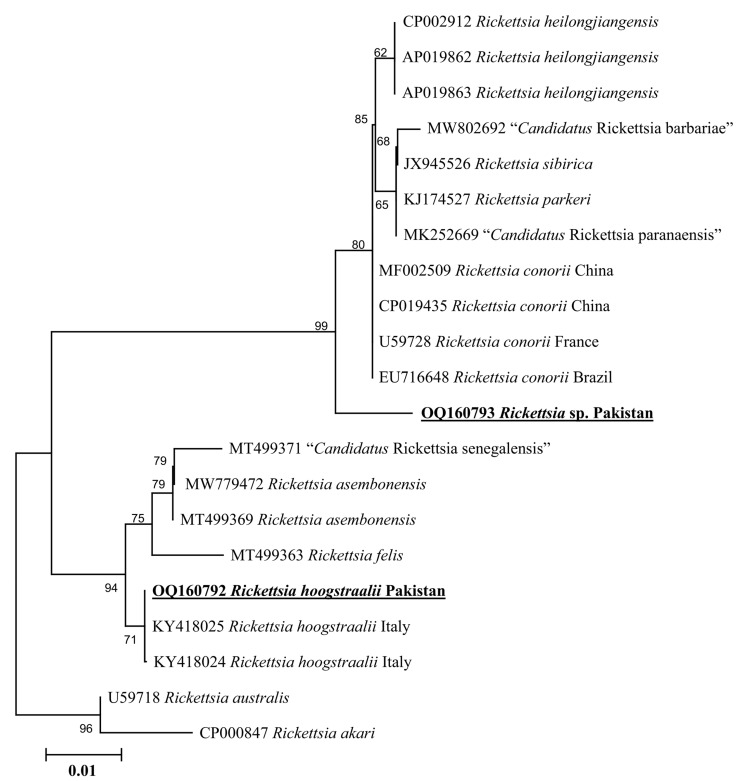
Neighbor-Joining phylogenetic tree based on *gltA* sequences of *Rickettsia* sp. and *R. hoogstraalii*. The *gltA* sequences of *Rickettsia australis* and *Rickettsia akari* were employed as an outgroup. All sequences have been denoted by their GenBank accession numbers, followed by species name and country name. The bootstrap values (1000-replications) are shown at each node. The sequences (OQ160793 and OQ160792) of the present study have been marked with bold and underlined fonts.

**Figure 6:**
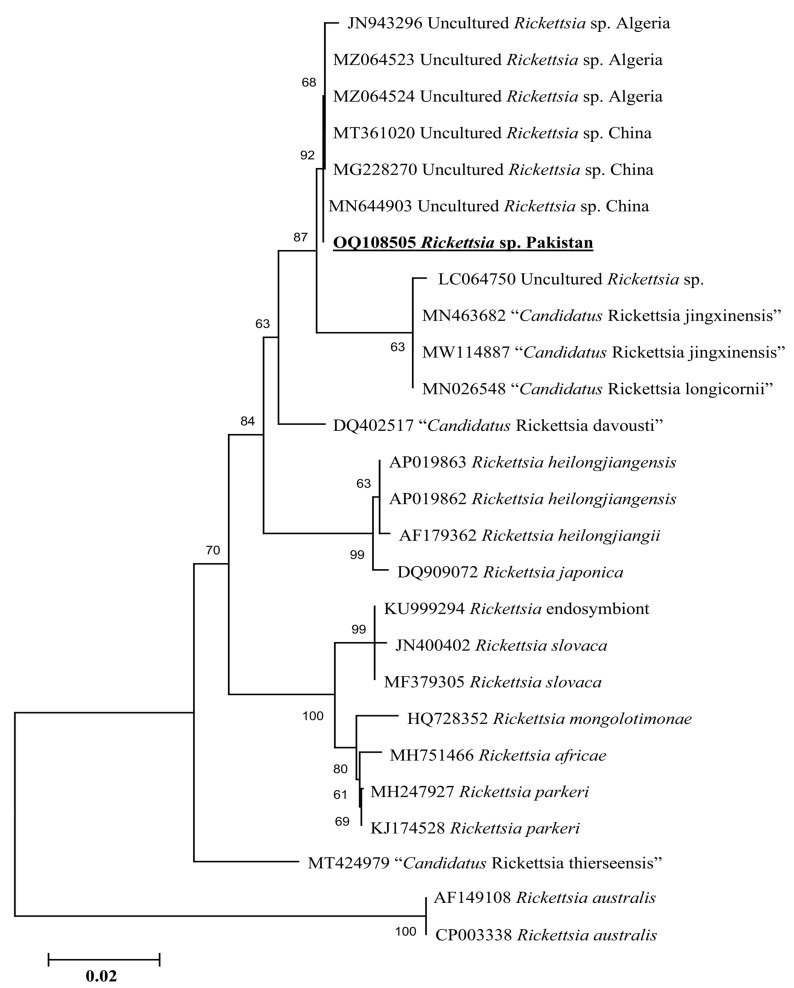
Neighbor-Joining phylogenetic tree based on *ompA* sequences of a *Rickettsia* sp. The *ompA* sequences of *R. australis* were employed as an outgroup. All sequences have been denoted by their GenBank accession numbers, followed by species name and country name. The bootstrap values (1000-replications) are shown at each node. The sequence (OQ108505) of the present study has been marked with bold and underlined fonts.

**Figure 7:**
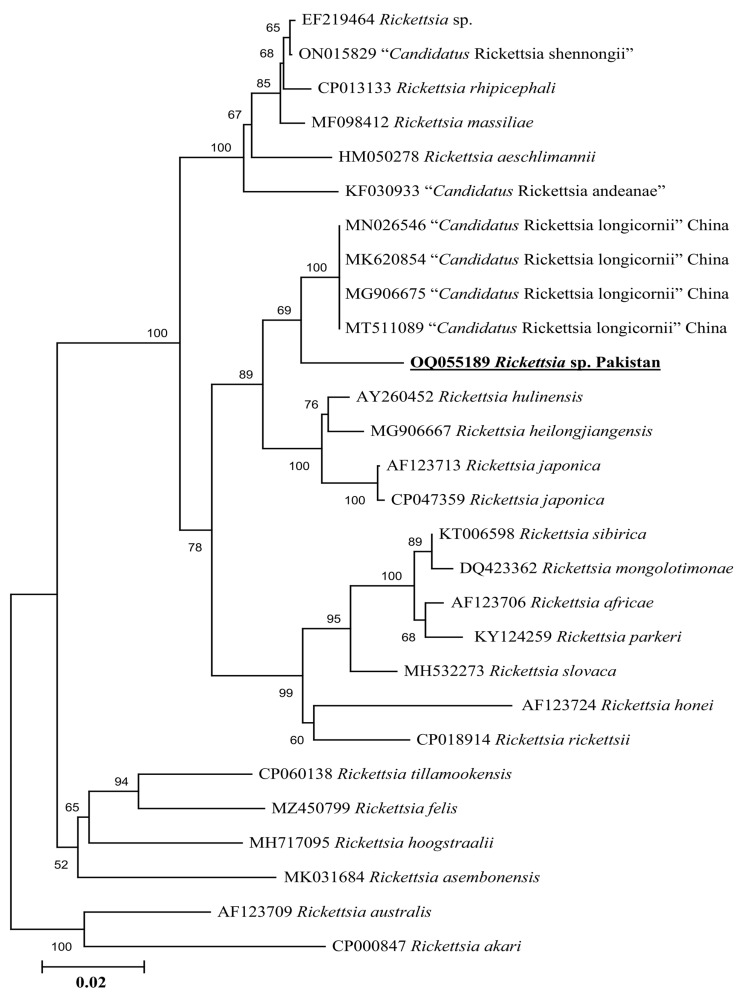
Neighbor-Joining phylogenetic tree based on *ompB* sequences of a *Rickettsia* sp. The *ompB* sequences of *R. australis* and *R. akari* were employed as an outgroup. All sequences have been denoted by their GenBank accession numbers, followed by species name and country name. The bootstrap values (1000-replications) are shown at each node. The sequence (OQ055189) of the present study has been marked with bold and underlined fonts.

Phylogenetic tree based on rickettsial *gltA*, the *Rickettsia* sp. detected in *H. kashmirensis* clustered individually with the sequence of *Rickettsia* spp. of the spotted fever group. In contrast, the *R. hoogstraalii* detected in *H. montgomeryi* was clustered with the same species reported from Italy (KY418024-KY418025) (Figure 5). The rickettsial *ompA* fragment detected in *H. kashmirensis* clustered with the *Rickettsia* sp. reported from China (MT361020, MG228270, and MN644903) and Algeria (MZ064523-MZ064524 and JN943296) (Figure 6) and grouped in a sister clade with “*Candidatus* Rickettsia longicornii” and “*Candidatus* Rickettsia jingxinensis”, while the rickettsial *ompB* fragment clustered with “*Candidatus* Rickettsia longicornii” reported from China (MN026546, MK620854, MG906675, and MT511089) (Figure 7). 

## 4. Discussion

As the geo-climatic conditions of the Oriental region including Pakistan suit the flourishment of *Haemaphysalis* ticks, the largest diversity of these ticks has been reported in this region [16]. The second most diverse genus of hard ticks (Ixodidae), *Haemaphysalis* comprises ~173 tick species globally. In Pakistan, 13 *Haemaphysalis* spp. have been reported; however, genetic data of these ticks are limited [16]. Despite the huge diversity, genetic data regarding the genus *Haemaphysalis* ticks and associated *Rickettsia* spp. have been largely neglected. For this purpose, *Haemaphysalis* ticks were collected from goats and sheep in northern Pakistan, where several species of this genus are considered endemic [16,18,19]. Herein, the collected ticks were morpho-molecularly identified as *H. cornupunctata, H. kashmirensis,* and *H. montgomeryi*. The genetic characterization based on 16S rDNA and *cox* partial sequences of *H. kashmirensis,* and *cox* sequence for *H. cornupunctata* was achieved for the first time. An undetermined *Rickettsia* sp. based on *gltA*, *ompA,* and *ompB* sequence was molecularly characterized in *H. kashmirensis,* whereas *R. hoogstraalii* based on only *gltA* sequence was detected in *H. montgomeryi*. 

The surveyed areas are part of the HKH mountain range, which has been considered as one of the most important biodiversity hotspots [18,33]. Previously, *H. kashmirensis*, *H. cornupunctata,* and *H. montgomeryi* have been reported from many locations of the HKH range, which spans different territories of the Indian subcontinent, including Pakistan [15,17,18], Kashmir and India [14], and Afghanistan [34]. These findings suggest that HKH mountain regions have a great diversity of the *Haemaphysalis* ticks owing to the abundance of suitable hosts and conducive climate conditions. 

*Haemaphysalis kashmirensis* was found less in number than *H. cornupunctata* and *H. montgomeryi,* which could be due to the association of the later species with hosts other than goats and sheep in the family Bovidae [1]. Moreover, the adult ticks (female and male) were outnumbered by the immature ticks (nymphs), while no larval stage of any tick species was found on goats and sheep. Previous studies suggested that *Agama tuberculata* (Kashmir Rock Agama) is the main host of the nymphal and larval stages of *H. kashmirensis,* which is found in the HKH range [21]. Similarly, animals belonging to the Muridae, Herpestidae, Erinaceidae, Cricetidae, and Soricidae families have been recorded as the main hosts for the nymphal and larval ticks of *H. cornupunctata* and *H. montgomeryi* [1].

*Haemaphysalis* ticks have been reported as vectors for *Rickettsia* spp. including *R. hoogstraalii* and *Rickettsia rhipicephali* [5,35]. Herein, *Rickettsia* sp. was detected through *gltA*, *ompA,* and *ompB*, whereas *R. hoogstraalii* was detected only through *gltA*. The genetic characterization of *R. hoogstraalii* was also attempted through *ompA* and *ompB*; however, the amplifications of these fragments were unsuccessful. Amplification failures are common in the case of *ompA*, *ompB* that might be the lack of targeted genes, as shown in the transition group *Rickettsia* or due to primer mismatching [36,37,38]. *Rickettsia hoogstraalii* has been detected in ticks of the genus *Haemaphysalis* such as *H. sulcata* (Cyprus and Italy), *H. punctata* (Italy), and *Haemaphysalis parva* (Turkey) [39,40,41]. The pathogenicity of *R. hoogstraalii* is poorly known [42]. 

Genetic analyses based on molecular markers such as 16S rDNA and *cox* genes are extremely helpful in unveiling the systematics of ticks and phylogenetic positioning [43,44,45,46,47,48,49]. In the phylogenetic tree based on 16S rDNA and *cox* of *H*. *cornupunctata*, this species appeared in a monophyletic branch with *H*. *punctata* from Turkey, France, China, Iran, Romania, Portugal, and Hungary. Previously, these two tick species (*H*. *cornupunctata* and *H. punctata*) have been assigned to the same subgenus (*Aboimisalis*) on a morphological basis [11]. In a phylogenetic tree based on 16S rDNA and *cox* of *H. kashmirensis*, this specie clustered in a monophyletic clade with *H. sulcata* previously reported from Pakistan, China, Turkey, France, and Iran. On a morphological basis, *H. sulcata* and *H. kashmirensis* have been placed in the same subgenus, *Herpetobia* [11]. A phylogenetic clustering of these species with different closest species of the same subgenus could be associated with the missing genetic data of corresponding species in the GenBank. The phylogenetic analysis based on rickettsial *gltA* showed that the *Rickettsia* sp. detected in *H. kashmirensis* belonged to the SF group, whereas *R. hoogstraalii* detected in *H. montgomeryi* belonged to the transition group. The phylogenetic tree based on *ompA* and *ompB*, obtained from the same sample in which *Rickettsia* sp. was detected, validated the *gltA*-based phylogenetic analysis for *Rickettsia* sp. 

## 5. Conclusions

This study contributes to the missing information regarding the genetic data of some *Haemaphysalis* ticks, especially *H. kashmirensis,* which was genetically characterized for the first time. The relationship of *H*. *cornupunctata* with subgenus *Aboimisalis* and *H. kashmirensis* with subgenus *Herpetobia* established on a morphological basis was confirmed through molecular-based phylogenetic analysis. Furthermore, a *Rickettsia* sp. was molecularly assessed in *H*. *kashmirensis*, whereas *R. hoogstraalii* was detected in *H. montgomeryi*. This study may assist in understanding the identification, evolutionary history, and molecular epidemiology of *Haemaphysalis* ticks and associated *Rickettsia* spp. Further studies should genetically characterize and evaluate the pathogenicity of these *Rickettsia* spp.

## Figures and Tables

**Table 1:** Primers and PCR conditions used in the current study.

Organisms/Genes	Sequence (5′-3′)	Amplicons Size	Annealing Temperatures	References
Ticks/*cox*	HCO2198: TAAACTTCAGGGTGACCAAAAAATCA	710 bp	49 °C	[23]
	LCO1490: GGTCAACAAATCATAAAGATATTGG			
Ticks/16S rDNA	16S+1: CCGGTCTGAACTCAGATCAAG T	460 bp	54 °C	[24]
	16S-1: GCTCAATGATTTTTTAAATTGCTGT			
*Rickettsia*/*gltA*	CS-78: GCAAGTATCGGTGAGGATGTAAT	401 bp	56 °C	[25]
	CS-323: GCTTCCTAAAATTCAATAAATCAGGAT			
*Rickettsia/ompA*	Rr190.70: ATGGCGAATATTTCTCCAAAA	532 bp	55 °C	[26]
	Rr190.701: GTTCCGTTAATGGCAGCATCT			
*Rickettsia*/*ompB*	120-M59: CCGCAGGGTTGGTAACTGC	862 bp	50 °C	[27]
	120-807: CCTTTTAGATTACCGCCTAA			

**Table 2:** Data on the number of ticks and hosts, and detection of *Rickettsia* spp. by polymerase chain reaction.

Districts	Host		Species	Life Stages			Total	Tick Subjected to PCR	PCR for *Rickettsia* spp.							
	Common Name	Number (Infested/ Total)		Females	Males	Nymphs			*Rickettsia* sp.				*R. hoogstraalii*			
									*ompA*	*ompB*	*gltA*	Total Positive Samples (Infection Rates)	*ompA*	*ompB*	*gltA*	Total Positive Samples (Infection Rates)
Bajaur	Goats, Sheep	5/8	*H. cornupunctata*	22	14	5	41	3M, 3F, 3N	0	0	0	0	0	0	0	0
	Goats, Sheep	4/7	*H. kashmirensis*	18	12	3	33	3M, 3F, 3N	2F	0	2N	4 (44.44%)	0	0	0	0
	Goats, Sheep	6/9	*H. montgomeryi*	25	28	6	59	3M, 3F, 3N	0	0	0	0	0	0	0	0
Dir Lower	Goats, Sheep	6/8	*H. cornupunctata*	35	26	5	66	3M, 3F, 3N	0	0	0	0	0	0	0	0
	Goats, Sheep	5/7	*H. kashmirensis*	28	14	4	46	3M, 3F, 3N	0	0	2N	2 (22.23%)	0	0	0	0
	Goats, Sheep	12/16	*H. montgomeryi*	81	47	11	139	3M, 3F, 3N	0	0	0	0	0	0	2F, 2M	4 (44.44%)
Dir Upper	Goats, Sheep	12/15	*H. cornupunctata*	74	35	14	123	3M, 3F, 3N	0	0	0	0	0	0	0	0
	Goats, Sheep	11/15	*H. kashmirensis*	45	29	12	86	3M, 3F, 3N	2F, 2N	2N	0	6 (66.66%)	0	0	0	0
	Goats, Sheep	12/16	*H. montgomeryi*	77	61	14	152	3M, 3F, 3N	0	0	0	0	0	0	2F, 2N	4 (44.44%)
Swat	Goats, Sheep	4/6	*H. cornupunctata*	14	11	3	28	3M, 3F, 3N	0	0	0	0	0	0	0	0
	Goats, Sheep	4/6	*H. kashmirensis*	11	12	3	26	3M, 3F, 3N	2M	3F	0	5 (55.56%)	0	0	0	0
	Goats, Sheep	5/7	*H. montgomeryi*	19	12	4	35	3M, 3F, 3N	0	0	0	0	0	0	0	0
Total		86/120 (71.3%)		449 (53.8%)	301 (36%)	84 (10%)	834	108	4F, 2M, 2N	2N, 3F	4N	17 (15.74%)	0	0	4F, 2M, 2N	6 (5.56%)

M = male, F = female, and N = nymph.

## Data Availability

All the relevant data are within the manuscript.
